# Two Metallothionein Genes in *Oxya chinensis*: Molecular Characteristics, Expression Patterns and Roles in Heavy Metal Stress

**DOI:** 10.1371/journal.pone.0112759

**Published:** 2014-11-12

**Authors:** Yaoming Liu, Haihua Wu, Lihua Kou, Xiaojian Liu, Jianzhen Zhang, Yaping Guo, Enbo Ma

**Affiliations:** 1 Institute of Applied Biology, Shanxi University, Taiyuan, Shanxi, 030006, China; 2 College of Life Science, Shanxi University, Taiyuan, Shanxi, 030006, China; Ghent University, Belgium

## Abstract

Metallothioneins (MTs) are small, cysteine-rich, heavy metal-binding proteins involved in metal homeostasis and detoxification in living organisms. In the present study, we cloned two MT genes (*OcMT1* and *OcMT2*) from *Oxya chinensis*, analyzed the expression patterns of the *OcMT* transcripts in different tissues and at varying developmental stages using real-time quantitative PCR (RT-qPCR), evaluated the functions of these two MTs using RNAi and recombinant proteins in an *E. coli* expression system. The full-length cDNAs of *OcMT1* and *OcMT2* encoded 40 and 64 amino acid residues, respectively. We found Cys-Cys, Cys-X-Cys and Cys-X-Y-Z-Cys motifs in *OcMT1* and *OcMT2*. These motifs might serve as primary chelating sites, as in other organisms. These characteristics suggest that OcMT1 and OcMT2 may be involved in heavy metal detoxification by capturing the metals. Two *OcMT* were expressed at all developmental stages, and the highest levels were found in the eggs. Both transcripts were expressed in all eleven tissues examined, with the highest levels observed in the brain and optic lobes, followed by the fat body. The expression of *OcMT2* was also relatively high in the ovaries. The functions of *OcMT1* and *OcMT2* were explored using RNA interference (RNAi) and different concentrations and treatment times for the three heavy metals. Our results indicated that mortality increased significantly from 8.5% to 16.7%, and this increase was both time- and dose-dependent. To evaluate the abilities of these two MT proteins to confer heavy metal tolerance to *E. coli*, the bacterial cells were transformed with pET-28a plasmids containing the *OcMT* genes. The optical densities of both the MT-expressing and control cells decreased with increasing concentrations of CdCl_2_. Nevertheless, the survival rates of the MT-overexpressing cells were higher than those of the controls. Our results suggest that these two genes play important roles in heavy metal detoxification in *O. chinensis*.

## Introduction

Metallothioneins (MTs) were first discovered as cadmium-binding proteins and isolated from horse kidneys in 1957 [1]. MTs are low-molecular-mass (<10 kDa), cysteine-rich proteins (15–30% of their amino acid contents) that lack aromatic residues, resulting in their optimal capacities for metal ion coordination. These high cysteine levels are necessary for the coordination of metal ions through the thiolate cluster as facilitated by the Cys-X-Cys and Cys-X-Y-Cys motifs, in which X can be any amino acid other than cysteine [2]. These types of metal-binding proteins have been widely found in all organisms, including bacteria, plants, invertebrates and vertebrates [3]–[5]. More complete information is available in recently published reviews [6]–[9]. Studies involving MTs have been performed in various fields, including toxicology, physiology and molecular and developmental biology [10].

MTs play an important role in zinc and copper homeostasis as well as in the detoxification of non-essential trace elements, such as cadmium (Cd) and mercury (Hg), because of their characteristic high cysteine levels [11]. MTs also aid in protecting cells from oxidative stress via the intracellular scavenging of free radicals [9], [12]. Because of their high affinities for heavy metals, the roles of MTs in the detoxification of heavy metals and in maintaining essential metal ion homeostasis within cells have been widely investigated [7], [13]. *Drosophila* metallothioneins play important roles in copper homeostasis as well as in the detoxification of cadmium [14]. Paul (2000) found that 99.0% of cadmium is present in the gut epithelium in the form of metallothionein-bound cadmium after exposing *O. cincta* to different cadmium concentrations [15]. Several pieces of evidence have indicated that MTs can also act as scavengers of free hydroxyl and superoxide radicals. MT synthesis can be induced by oxidative stress and hormonal stimuli similar to heavy metals [16]–[18]. MTs are mainly considered to involved in the protection against oxidative stresses and neuroprotective mechanisms [19].

A number of studies have reported the detailed molecular structure of MT by molecular sequencing and nuclear magnetic resonance spectroscopic analysis and have investigated the roles of MTs in the detoxification of heavy metals. However, these studies have focused primarily on mammalian and aquatic organisms and plants [9]. Few studies have examined the interactions between MTs and metal ions in Diptera and Collembola insects [20], [21]. Little is currently known regarding the molecular characteristics and functions of MT genes in Orthoptera insects, especially grasshohper *Oxya chinensis*.

*Oxya chinensis*, which is an agricultural pest, feeds on the leaves of gramineous plants, particularly rice, and inhabits rice-growing areas of China. Due to grasshopper behavior in the farmland ecosystems, heavy metals (such as cadmium) in the agricultural environment transfer into the bodies of the grasshoppers through the food chain. Previous studies performed by our laboratory have indicated that heavy metals can accumulate in *O. chinensis* through the food chain [22], [23]. Our previous research has also found that MT levels increase in *O. chinensis* when the grasshoppers feed on wheat leaves containing heavy metals (data unpublished). It has been difficult to clone the MT sequences based on only several conserved cysteines, and thus, the molecular characteristics of the MTs and their roles in the detoxification of heavy metals have not been further studied. However, two MT sequences have been recently described in the *O. chinensis* transcriptome database, allowing additional analyses to be performed.

The present study aimed to 1) clone and identify two full-length cDNAs of MT genes from *O. chinensis*, 2) analyze the expression patterns of these two MT genes in different tissues and at different developmental stages, 3) investigate the functions of these two MT genes by RNAi, and 4) evaluate the tolerances of the two MT proteins to Cd using recombinant MT in an *E. coli* expression system. The present study will help to elucidate the characteristics and functions of MTs in *O. chinensis* and demonstrate the potential value of heavy metal pollution prevention.

## Materials and Methods

### Insects

*Oxya chinensis*, which is an important agricultural pest, inhabits a wide range of rice-growing areas spanning most of China. The *O. chinensis* used in this study were collected from paddy fields in the Jinyuan District, Taiyuan, Shanxi province (north latitude: 34.28, east longitude: 112.45) where there is no land protection of any type. Local farmers must use pesticides to kill these grasshoppers. We explicitly confirmed that no specific permissions were required for these locations/activities and that the field studies did not involve endangered or protected species.

The *O. chinensis* eggs were incubated in a climate chamber (Yiheng Co., Ltd. Shanghai, China) at 28±2°C with a 14:10-h (light: dark) photoperiod at 60–75% humidity. After hatching, the nymphs were raised in nylon net cages, and all grasshoppers were reared using fresh wheat leaves. Healthy and uniform sets of insects were selected for our experiments.

### Identification and sequencing of two *OcMT* cDNA fragment*s*

Two cDNA sequences were obtained from the *O. chinensis* transcriptome database from samples that included 1st-5th instar nymphs, adults, and cadmium-treated insects. Two full-length cDNA sequences were identified using BLASTX and were designated as *OcMT1* and *OcMT2*. To confirm the predicted coding sequences of these two genes, two specific primers were used to amplify the cDNAs by reverse transcription PCR (RT-PCR) using cDNA templates prepared from the whole insect body. The RT-PCR products were run on a 1.5% agarose gel, purified using a Gel Extraction Kit (Omega, Doraville, CA, USA) and subcloned into the pEASY-T3 Cloning Vector (TransGen Biotech Co., Ltd. Beijing, China) and then sequenced by GBI Biotechnology Co., Ltd. (Beijing, China).

The physical and chemical properties of OcMT were analyzed using the ExPASy online tools (http://us.expasy.org/tools). The similarities and characteristics of the two OcMTs were compared with those of other known insect species on the basis of the deduced amino acid sequences. The amino acid features were analyzed using the online BLAST program provided by NCBI (http://blast.ncbi.nlm.nih.gov/Blast.cgi).

### Total RNA extraction and cDNA synthesis

Total RNA was isolated from the liquid nitrogen-preserved samples using RNAiso Plus (TaKaRa, Dalian, China) according to the manufacturer's protocol. The RNA purity was estimated using a NanoDrop 2000 UV-Vis Spectrophotometer (Thermo, Waltham, MA, USA) according to the absorbance ratio of A260/280, and its integrity was assessed by 1.5% agarose gel electrophoresis. One microgram of RNA was used to synthesize the first-strand cDNA using M-MLV Reverse Transcriptase (Promega, Madison, WI, USA) and an oligo-(dT) 18 primer.

### Expression patterns of *OcMT1* and *OcMT2* at the developmental stages and in the tissues

To determine the expression patterns of the *OcMT1 and OcMT2* genes at the seven developmental stages, including the egg, first-, second-, third-, fourth-, and fifth-instar nymphs and the adults, insects at day 3 of each stage were collected for total RNA extraction. To detect the tissue-dependent expressions of *OcMT1* and *OcMT2*, eleven selected tissues were dissected from the adults (pooled from ten adults) under a binocular microscope, including brain, optic lobe, muscle, foregut, midgut, hindgut, gastric caeca, Malpighian tubule, fat body, testis and ovary tissues.

RT-qPCR was conducted in a 20-µL reaction containing 2 µL of 20-fold diluted cDNA, 0.8 µL of each primer, 6.4 µL distilled water and 10 µL SYBR Green Real-time Master Mix (TOYABO, Japan) using the Applied Biosystems 7300 Real-time PCR System (Applied Biosystems, USA). *β-actin* was used as the reference gene. The optimized RT-qPCR program that was used for both *β-actin* and the OcMTs consisted of an initial step at 95°C for 15 s followed by 40 cycles of 95°C for 15 s and 60°C for 34 s. A melting curve was evaluated for each RT-qPCR experiment to confirm the amplification efficiency. All experiments were performed in triplicate, each with two technical replicates. Amplification specificity was verified using the dissociation curve. The fold changes for comparing the relative gene expression levels to those of the controls in the different tissues and at the different developmental stages were determined using the 2^−ΔΔCt^ method [24]. The sequences of the primers used for the RT-qPCR analysis are shown in Table 1.

**Table 1:** List of primers used in this study.

Application of primers	Gene name	Primer sequence (5′-3′)	Product size (bp)
**cDNA cloning**	***OcMT1***	**F: GTTGCTGAAGCCGCCTACT**	**172**
		**R:CATCTTGGGTGGCTGGTG**	
	***OcMT2***	**F: CCGCTCTGACAAGCAGGAAC**	**259**
		**R: CTGCCTGGTGATCTATGGGT**	
**RT-qPCR analysis**	***OcMT1***	**F: GTTGCTGAAGCCGCCTACT**	**172**
		**R: CATCTTGGGTGGCTGGTG**	
	***OcMT2***	**F: ATGTCGTCTCCGTGCTGT**	**123**
		**R: GCCCTTTGTTTCCTCCTT**	
	***β-*** **actin**	**F: CGAAGCACAGTCAAAGAGAGGTA**	**156**
		**R: GCTTCAGTCAAGAGAACAGGATG**	
**dsRNA synthesis**	***OcMT1***	**F:TAATACGACTCACTATAGGG GCTGAAGCCGCCTACTTCTA**	**169**
		**R: TAATACGACTCACTATAGGG CATCTTGGGTGGCTGGTG**	
	***OcMT2***	**F:TAATACGACTCACTATAGGG CGCTCTGACAAGCAGGAACT**	**193**
		**R:TAATACGACTCACTATAGGG ATCGTCTCCCTGTTTGCACT**	
	***GFP***	**F: TAATACGACTCACTATAGGG GTGGAGAGGGTGAAGG**	**712**
		**R: TAATACGACTCACTATAGGG GGGCAGATTGTGTGGAC**	
**Heterologous gene expression**	***OcMT1***	**F: GTGGGATCCATGCCTGACCCGTG**	**141**
		**R:ACGAAGCTTTCACTTAGAGGTGGT**	
	***OcMT2***	**F: GTGGGATCCATGTCGTCTCCGTGC**	**213**
		**R:ATTAAGCTTTCATTCACATTTGCAGC**	

### Functional analysis of *OcMT1* and *OcMT2* by RNAi

To evaluate their vital biological functions, an RNA interference analysis of both the *OcMT1* and *OcMT2* genes was performed by injecting sequence-specific double-stranded RNA (dsRNA) into *O. chinensis*. The specialized PCR was performed using cDNA from the whole bodies of the adults to prepare the templates for the *OcMT1* and *OcMT2* dsRNA syntheses. The primers used for the synthesis of the dsRNA and the transcript analysis are shown in Table 1. The PCR products of *OcMT1* and *OcMT2* were subcloned and sequenced to confirm their specific identities. The *OcMT1* and *OcMT2* dsRNA and *GFP* were prepared and synthesized using the T7 RiboMAX Express RNAi System (Promega, Madison, WI, USA) following the manufacturer's instructions. The prepared dsRNA was dissolved in nuclease-free water, and a product contained within a single band was verified using a 1.5% agarose gel. The concentration of dsRNA was adjusted to 1.5 µg.µL^−1^. Aliquots of 4 µL of the *OcMT1* and *OcMT2* dsRNA (containing 6 µg dsRNA) were injected into the abdomens between the second and third abdominal segments of the adult insects using a manual microinjector (Ningbo, China). The control groups were injected with equivalent volumes of ds*GFP* alone. All experiments were performed in triplicate.

The whole bodies of three adults from each replicate were pooled for total RNA extraction at 12, 24 and 48 h after the injections of ds*OcMT1*, ds*OcMT2* and ds*GFP*, respectively. The efficiencies of the RNA silencing of the two *MT* genes were evaluated by measuring their mRNA transcription levels using RT-qPCR as described in Section 2.4.

To evaluate the roles of *OcMT1* and *OcMT2* in the detoxification of heavy metals, five concentration gradients of CdCl_2_ (0.87, 1.74, 2.61, 3.48, 4.35 mM), CuCl_2_ (8.79, 11.73, 14.67, 17.61, 20.55 mM) and ZnSO_4_ (15.65, 19.13, 22.61, 26.09, 29.57 mM) were prepared. Injections of ds*OcMT1*, *OcMT2 and* ds*GFP* dsRNA was administered, and after 24 h, 4 µL of the heavy metal solutions were injected into the abdomens of the insects. Each replicate contained 50 insects, and the experiments were performed using three replicates. The mortalities for each group were measured at 48 h after the heavy metal injections.

### Recombinant expressions of OcMT1 and OcMT2 in *Escherichia coli*

To further determine the roles of OcMT1 and OcMT2 in heavy metal detoxification, we constructed a recombinant plasmid that produced the OcMT1 and OcMT2 proteins in an *E. coli* expression system. The coding sequences of *OcMT1* and *OcMT2* were obtained by PCR amplifications with primers (Table 1) containing BamHI and HindIII sites, and the products were then digested with these two restriction endonucleases. The resulting digests were ligated into the BamHI and HindIII sites of the expression vector pET-28a, in which OcMT was expressed under the T7 bacteriophage promoter. The recombinant plasmids, pET-28a-OcMT1 and pET-28a-OcMT2, were transformed into *E. coli* DH5a-competent cells and were then sequenced (Invitrogen China Limited, Shanghai, China). The transformation mixture was plated onto LB agar, containing 100 µg mL^−1^ kanamycin. One positive clone for each cDNA was transformed into competent *E. coli* BL21 (DE3) cells for the expressions of the proteins. The OcMT fusion protein was expressed in *E. coli* and induced with 1 mM isopropyl b-D-thiogalactoside (IPTG) for 6 h at 33°C. A parent vector lacking an OcMT gene insert was used as a negative control. The cells were harvested by centrifugation at 12,000 g for 10 min. The cells were then lysed by mild sonication at 4°C and centrifuged at 12,000 g for 15 min, and the fusion proteins were isolated from the supernatants. The fractions in the crude BL21 (DE3) cell lysates harboring pET-28a-OcMT1 and pET-28a-OcMT2 were detected by 15% SDS-PAGE.

### Evaluation of heavy metal tolerance using recombinant OcMTs

To further investigate the metal tolerance of the transformed *E. coli* BL21 (DE3) cells, 5 mL of each culture at OD_600_ = 0.6 was inoculated into new tubes, containing 5 mL of liquid nutrient LB medium. The tubes were shaken at 37°C until the OD_600_ measurements were between 0.50 and 0.55. The cells containing pET-28a-OcMT were divided into two groups. One group was induced by 1 mM IPTG, and the other was not. A parent vector (pET-28a) without inserts was used as a negative control. The desired concentrations of CdCl_2_ (0, 0.82, 1.64 and 3.27 mM) were added to the cells containing pET-28a-OcMT and the pET-28a control vector in the 5-mL cultures (LB medium plus kanamycin). Bacterial growth was monitored by measuring the optical densities of the cultures at 600 nm using a SpectraMAX 190 (Molecular Devices, California, USA) at 1-h intervals. Three independent experiments with three replicates for each concentration were performed.

### Data analysis

The MT mRNA levels in the different tissues and at the various developmental stages and the mortalities between the control and the exposed groups were analyzed using a one-way ANOVA followed by Tukey's HSD test. Differences were considered statistically significant at *P*<0.05. All data are shown as the mean ± standard deviation, and the statistical analyses were performed using the SPSS version 11.5 software (SPSS Inc., Chicago, IL, USA).

## Results

### Analysis of cDNAs and deduced amino acid sequences of *OcMTs*

**Figure 1:**
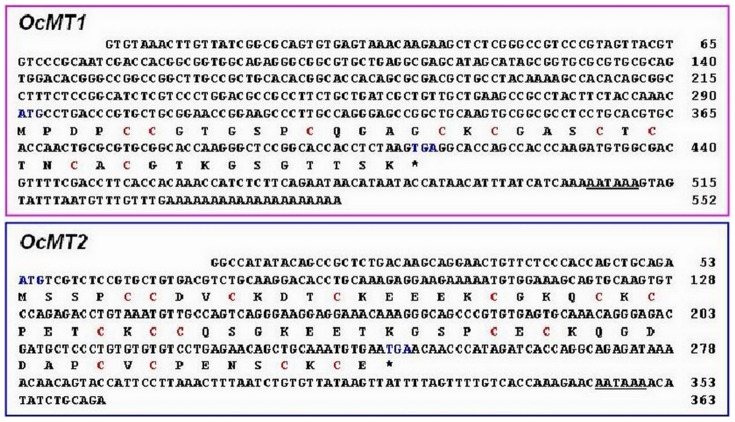
Nucleotide and deduced amino acid sequences of *OcMT1* and *OcMT2* cDNAs from *Oxya chinensis*. The deduced amino acid sequence is shown below the nucleotide sequence. Blue letters indicate the start codon (ATG), and an asterisk (*) indicates the stop codon. The putative polyadenylation signal sequence (AATAAA) is underlined. The numbers on the right refer to the amino acid residues. The cysteines (C) are highlighted in red. The deduced amino acid sequences of *OcMT1* and *OcMT2* are shown, with the cysteine residues arranged as C-C, C-X-C and C-X-X-C motifs, in which X can be any amino acid other than cysteine.

**Figure 2:**
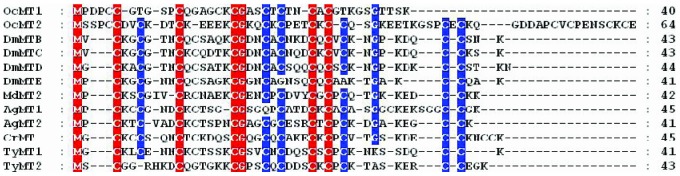
Multiple sequence alignments of the deduced amino acid sequences of *OcMT1* and *OcMT2* with other known homologues species using the GeneDoc software. Amino acid residues are shaded in color. Conserved and identical cysteine amino acids are highlighted in red and blue, respectively. The species and the GenBank accession numbers are as follows: *Oxya chinensis* (OcMT1 KJ153014 and OcMT2 KJ153015), *Drosophila melanogaster* (DmMTB: NP524413, DmMTC: NP650882, DmMTD: NP788695, and DmMTE: NP001189254) *Musca domestica* (MdMT2: AEO50699), *Anopheles gambiae* (AgMT1: AAX86006 and AgMT2: AAX86007), *Chironomus riparius* (CrMT: ADZ54163), *Tabanus yao* (TyMT1: ABX80078 and TyMT2: AAX860079).

Two full-length cDNA sequences were obtained from the *O. chinensis* transcriptome database putatively encoding two different *OcMTs*, which were named *OcMT1* and *OcMT2* (GenBank accession numbers KJ153014 and KJ153015) (Fig. 1). The full-length cDNA sequence of *OcMT1* was 552 base pairs (bp) long, with an open reading frame of 123 bp that encoded a 40-amino acid peptide with a predicted molecular weight (MW) of approximately 3.74 kDa. *OcMT1* had a theoretical isoelectric point (pI) of 8.11 and one Cys-Cys and three Cys-X-Cys motifs (CCXXXXXCXXXXCKCXXXCTCTNCAC). The full-length cDNA sequence of *OcMT2* was 363 bp, with an open reading frame (ORF) of 195 bp that encoded a 64-amino acid peptide. The predicted peptide molecular mass and isoelectric point (pI) were 6.92 kDa and 4.88, respectively, according to the ExPASy Proteomics website. OcMT2 contained two Cys-Cys, three Cys-X-Cys and three Cys-X-Y-Z-Cys motifs. One Cys-Cys and three Cys-X-Y-Z-Cys motifs (CCDVCXXXCKEEEKCXXXCKCXXXCK) were at the N terminus, and one Cys-Cys and three Cys-X-Cys motifs (CCQSGKEETKGSPCECKQGDDAPCVCPENSCKCE) were at the C terminus, which is a structure typical of MT proteins. The cysteine concentrations of the deduced OcMT1 and OcMT2 protein sequences were 22.5% and 25%, respectively (Fig. 2). Two OcMT sequences contained 9 and 16 cysteines, respectively, and all cysteine residues were in the characteristic Cys-Cys, Cys-X-Cys, Cys-X-Y-Cys or Cys-X-Y-Z-Cys configuration similar to that observed in other MTs. The polyadenylation signal (AATAAA) was located upstream of the poly (A) tract. The deduced amino acid sequences of the MTs from *O. chinensis* were compared with the other insect MTs using the GeneDoc FASTA sequence comparison program. As shown in Fig. 2, the two OcMTs shared low amino acid sequence similarities but high identities. Importantly, they were found to code for conserved cysteine residues and functional motifs, such as Cys-Cys, Cys-X-Cys and Cys-X-Y-Cys, that are found in other species.

### Tissue expression patterns of *OcMT1* and *OcMT2*

**Figure 3:**
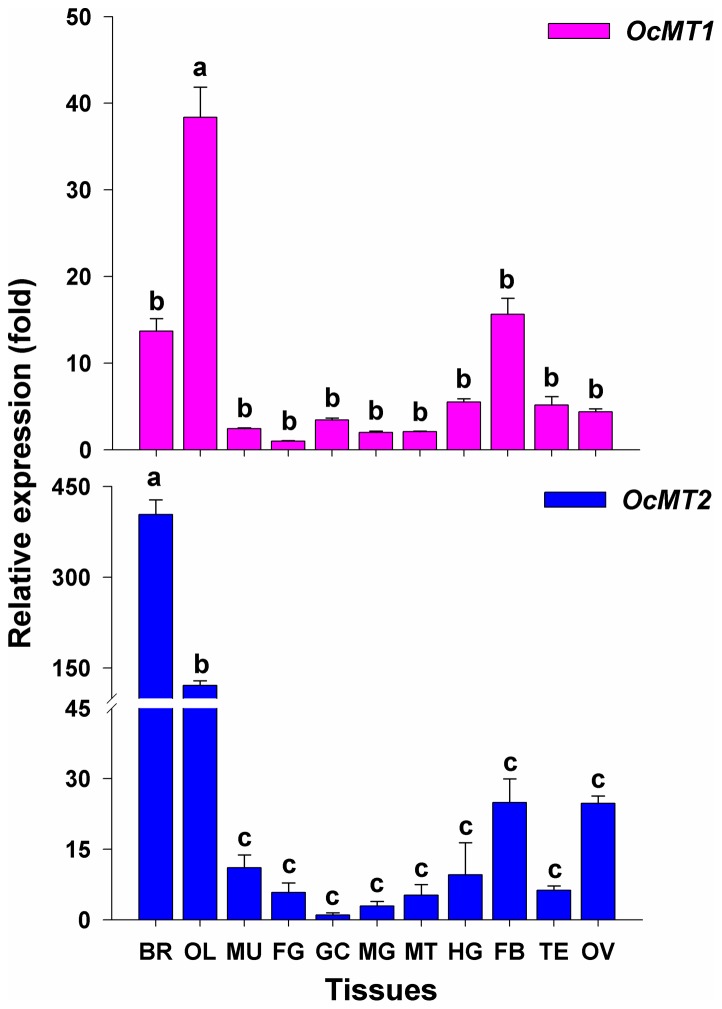
Tissue-specific transcript expressions of *OcMT1* and *OcMT2* in adults on day 3. The *β-actin* gene was used as an internal control. The tissues include the brain (BR), optic lobe (OL), muscle (MU), foregut (FG), midgut (MG), hindgut (HG), gastric caeca (GC), Malpighian tubule (MT), fat body (FB), testis (TE) and ovary (OV). The different letters on the error bars indicate significant differences in the expression of the same gene in different tissues. The data are expressed as the means ± SE of three biological replicates. The relative expressions of *OcMT1* and *OcMT2* were calculated using 2^−ΔΔCt^. The vertical bars represent the mean ± SE. (*P*<0.05, Tukey's HSD test; n = 3).

The RT-qPCR analysis indicated that *OcMT1* mRNA was widely expressed in all tissues examined. Its expression levels were highest in the optic lobes, which exhibited 2.5- and 3-fold higher levels compared with those of the fat bodies and brain, respectively. The *OcMT1* transcript levels found in the optic lobe were 7- to 30-fold higher compared with all other tissues examined (Fig. 3). The highest *OcMT2* expression levels were detected in the brain, and high levels were also found in the optic lobes; however, low levels were observed in the muscles, foregut, midgut, hindgut, gastric caeca, Malpighian tubules, fat bodies, testes and ovaries. The *OcMT2* expression levels in the brain were approximately 5- to 350-fold higher compared with the other tissues.

### Stage-dependent expression patterns of *OcMT1* and *OcMT2*

**Figure 4:**
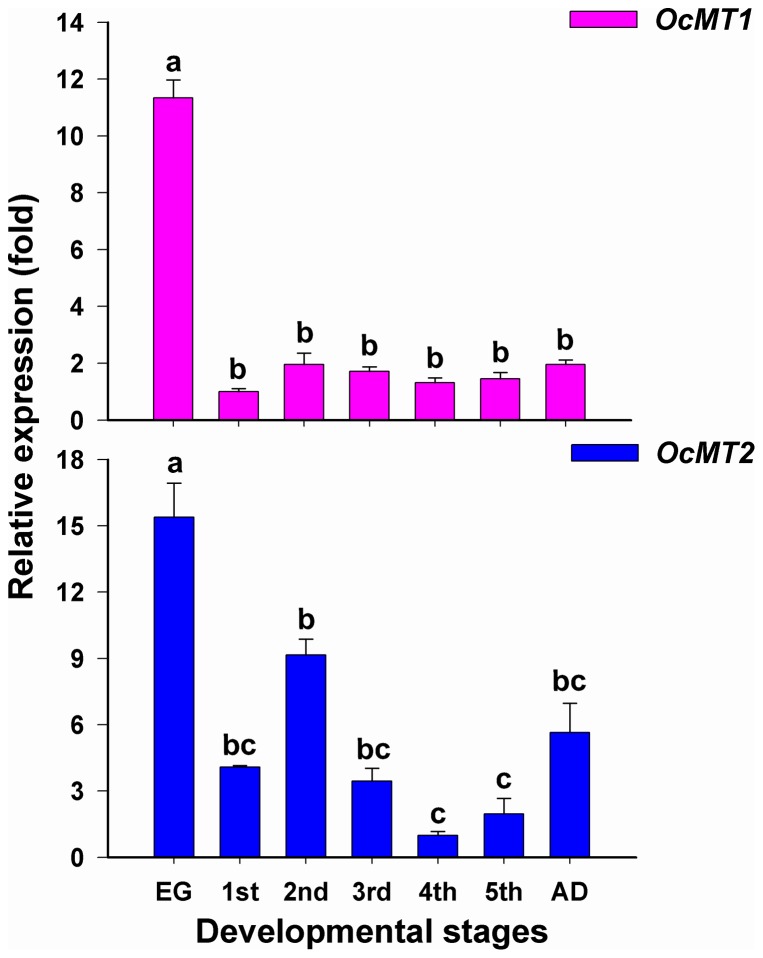
Analysis of stage-dependent expressions of *OcMT1* and *OcMT2* in *O. chinensis* by RT-qPCR. The same letters on the error bars indicate no significant differences in the expression of the same gene in the seven developmental stages. The relative expression of *OcMT* gene was calculated using 2^−ΔΔCt^. The vertical bars represent the mean ± SE. (*P*<0.05, Tukey's HSD test; n = 3).

The relative mRNA expression profiles of the *OcMT1 and OcMT2* genes indicated that their expression levels varied significantly throughout the seven life stages (Fig. 4). The highest expression levels of *OcMT1* were detected at the egg stage (3.5- to 8-fold higher than in the other stages), and the lowest levels were observed at the 1st-instar nymph stage. *OcMT2* displayed the lowest expression levels at the 4th-instar nymph stage and the highest levels at the egg stage (1.5- to 7-fold higher than in the remaining stages).

### Functional analysis of *OcMT1* and *OcMT2* by RNAi

**Figure 5:**
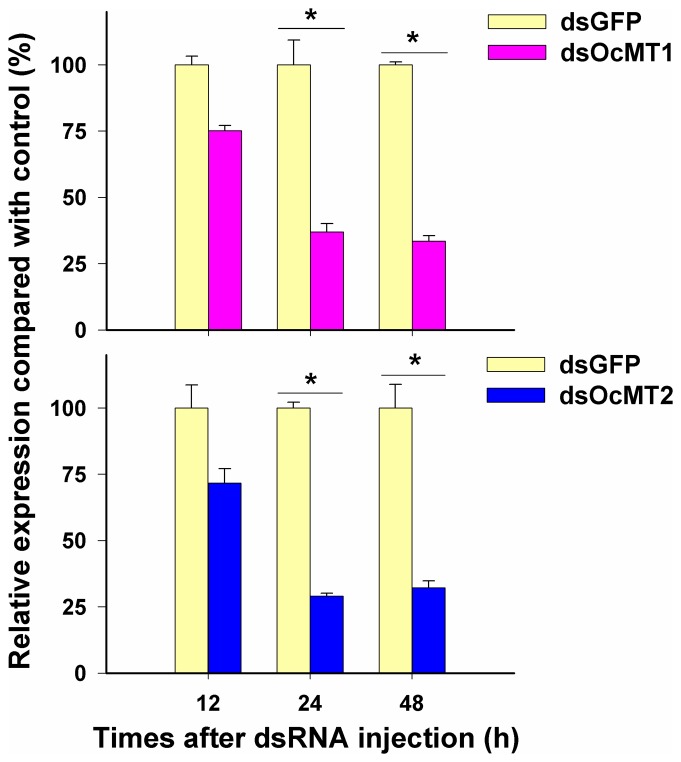
Efficiency of RNAi of *OcMT1* and *OcMT2*. Insects were injected with *OcMT1* and *OcMT2* dsRNA, and the control group was injected with dsGFP. The expression of *OcMT1* and *OcMT2* in the whole body was detected by RT-qPCR after 12, 24, 48 h of treatment. The expression levels of *OcMT1* and *OcMT2* mRNA for the control groups were set as 100%. An asterisk (*) on the error bars indicates significant differences (*P*<0.05, n = 3).

**Figure 6:**
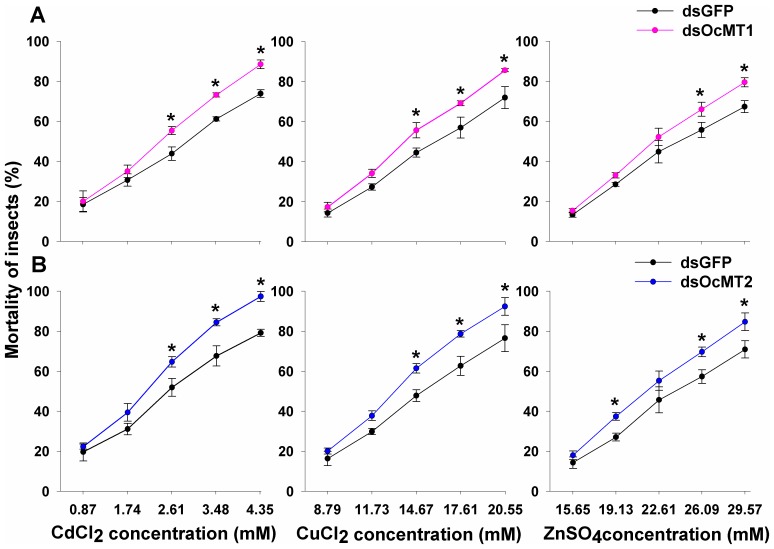
Mortalities of *O. chinensis* injected with different heavy metals after *OcMT1* and *OcMT2* gene silencing by RNAi. A: *OcMT1* RNAi; B: *OcMT2* RNAi. Insects were injected *with OcMT1* and *OcMT2* dsRNA, and the control groups were injected with the same amount of ds*GFP*. After 24 h, at least 100 insects were randomly selected, and 4 µL of a concentration gradient of CdCl_2_ (0.87, 1.74, 2.61, 3.48, 4.35 mM), CuCl_2_ (8.79, 11.73, 14.67, 17.61, 20.55 mM) and ZnSO_4_ (15.65, 19.13, 22.61, 26.09, 29.57 mM) solution were injected. The control groups were injected with distilled water. Mortalities were recorded at 48 h after the injections of the metal solutions. An asterisk (*) on the error bars indicates significant differences (*P*<0.05, Tukey's HSD test; n = 3).

After the injections of the dsRNAs, the *OcMT1* and *OcMT2* transcript levels in the whole bodies of the adults decreased by approximately 63.1% to 70.9% by 24 h and 48 h post-injection, but no significant differences were observed at 12 h (Fig. 5). As shown in Fig. 6, when *OcMT1* was silenced at 48 h, the mortalities increased from 64% to 72.5% for CdCl_2_, from 72.3% to 83.7% for CuCl_2_, and from 69% to 79.5% for ZnSO_4_. Similarly, the mortalities of the grasshoppers increased from 80.5% to 97.2% for CdCl_2_, from 76.5% to 91.5% for CuCl_2_, and from 70.9% to 84.7% for ZnSO_4_ after *OcMT2* was silenced. The mortalities of each group displayed dose-dependent increases of 8.5% to 11.4% and 13.8% to16.7% after the silencing of *OcMT1* and *OcMT2*, respectively.

### Roles of OcMTs in heavy metal tolerance as determined using recombinant proteins

**Figure 7:**
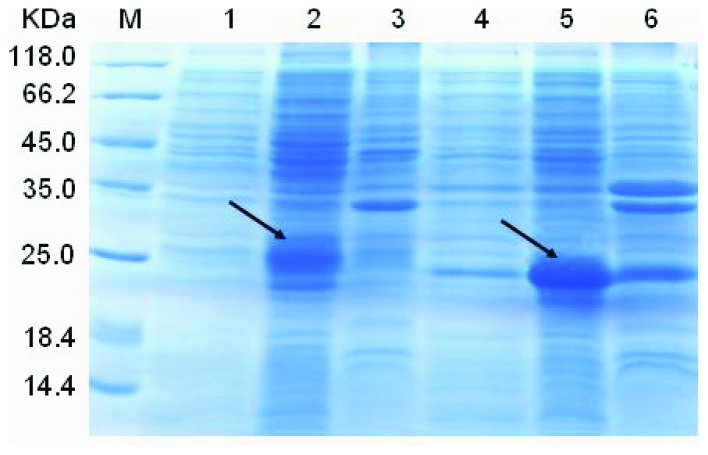
SDS-PAGE analysis of His and two His-OcMT fusion proteins expressed in *E. coli* BL21 (DE3) cells. Protein samples were separated by 15% SDS-PAGE and stained with Coomassie brilliant blue. Lane 1, medium range molecular weight marker; Lane 2, *E. coli* BL21 with pET-28a-OcMT2 cell lysate induced with IPTG; Lane 3, *E. coli* BL21 with pET-28a-OcMT2 without IPTG; Lane 4, *E. coli* BL21 with pET-28a; Lane 5, *E. coli* BL21 with pET-28a-OcMT1 cell lysate induced with IPTG; Lane 6, *E. coli* BL21 with pET-28a-OcMT1 without IPTG induction.

**Figure 8:**
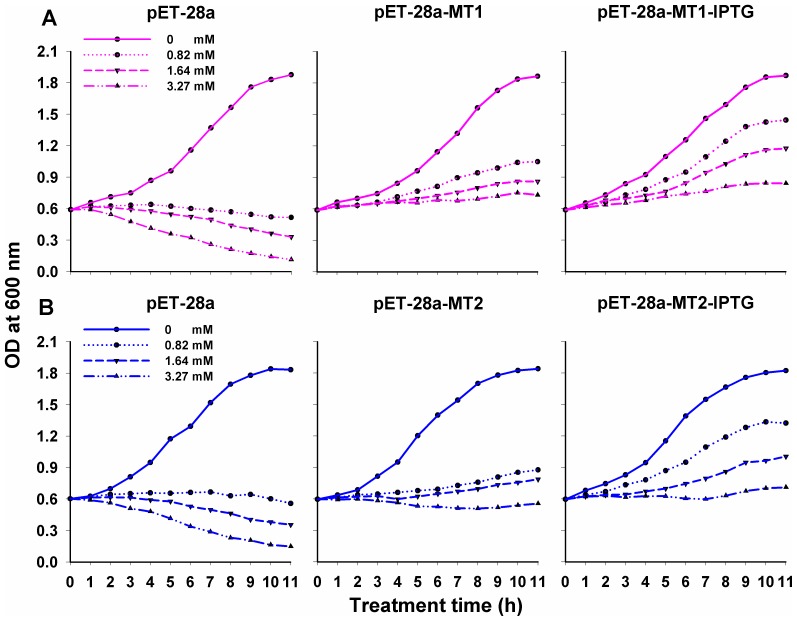
Cadmium tolerance of *E. coli* BL21 cells expressing *OcMT1* and *OcMT2*. A: OcMT1, B: OcMT2. Bacterial growth curve of *E. coli* cells transformed with pET-28a, pET-28a-OcMT and pET-28a-OcMT-IPTG. pET-28a is an “empty” vector; pET-28a-OcMT group is transformed with the *OcMT1* or *OcMT2* gene without IPTG; pET-28a-OcMT-IPTG group is transformed with the *OcMT1* or *OcMT2* gene with IPTG. Five microliters of CdCl_2_ was added into medium when bacteria were grown to OD600 = 0.6. All bacteria were grown for 11 h. Concentration gradient of CdCl_2_ were 0, 0.82, 1.74, 3.27 mM.

OcMT1 and OcMT2 were expressed successfully as shown in Fig. 7, lanes 2 and 5. The theoretical molecular weights of OcMT1 and OcMT2 are 3.74 and 6.92 kDa, respectively. Our results indicated that the OD values of the BL21(DE3) cells (pET-28a-MT1-IPTG group) were 1.37–2.82-fold higher than those of pET-28a-MT1 and pET-28a, and the OD values of the BL21(DE3) cells (pET-28a-MT2-IPTG group) were 1.15–3.92-fold higher than those of pET-28a-MT2 and pET-28a (Fig. 8). The OD values of the BL21 (DE3) cell pET-28a-MT1/2 strains were higher than those of the pET-28a groups, which may have been due to leaky expression caused by the presence of beta-galactosidase in the liquid nutrient LB medium.

## Discussion

There have been several reports of MTs in various species, including insects and animals. The numbers of MTs vary among different species. For example, *Drosophila melanogaster* has five MTs [21], but only a single MT has been identified in *Orchesella cincta* [15]. In the present study, two full-length MT cDNA sequences were obtained from the *O. chinensis* transcriptome database. These two MTs possessed different coding sequences, peptides, and cysteine concentrations. In particular, the amino acid sequence of the OcMT1 protein was similar in length to those of the metallothioneins (MTA, MTB, MTC and MTD) in *Drosophila*, which vary from 40 aa to 44 aa [25] and are much shorter than the MTs of most other species, which range in size from 58 aa to 61 aa.

Importantly, both the *OcMT1* and *OcMT2* transcripts code for most of the conserved cysteine residues and functional motifs (such as C-C, C-X-C and C-X-Y-C) that are typical of metallothionein structures. The conserved structural patterns are CCX_(5)_CX_(4)_CXCGASCXCTNCXC X_(10)_ in OcMT1 and CCXXCKDTCKX_(4)_CGKQCKCPETCK at the N terminus and CCX_(11)_CECX_(7)_CVCX_(4)_CKC at the N terminus and the C terminus of OcMT2. The non-cysteine-rich spacer region between the two termini has been proposed to play important roles in the stabilization and subcellular localization of MTs [26]. A total of 16 cysteine residues were found along the entire OcMT2 sequence, and cysteine and lysine (Lys, K) were adjacent at four positions. The Cys residues adjacent to Lys have been suggested to play roles in the structures and stabilities of the metal-binding sites of the protein [27]. These important structural characteristics suggest that OcMT1 and OcMT2 may be involved in heavy metal detoxification by capturing the metal within the tissues and that these residues may serve as primary chelating sites [28], [29].

A previous study has reported high MT protein levels in the nervous systems of grasshoppers (data not published). In this study, the OcMT mRNA levels were very highly expressed in the brain and optic lobe. This may suggest that the MTs in insects are the most responsive to harmful environmental stresses and are associated with neuroprotective mechanisms. There is no information available regarding the neuronal distribution of the MTs in insects. Studies of MT expression in the nervous system have been focused on humans, model animals and aquatic organisms. In mammals, three major MT isoforms are expressed widely throughout the adult central nervous system [30], [31]. MTs have been consistently found to be upregulated in mammalian brains in which neuroinflammation and oxidative stress are present, for example, in cases of acute or chronic brain injury [32], [33]. These studies concluded that MTs have important functions in the central nervous system and brain because MT-1 and MT-2 protect the central nervous system from damage induced by chemical and physical injuries [34], [35].

We found that two *OcMT* genes were widely expressed in the digestive tissues (FG, GC, MG and HG). These results are consistent with previous findings. In other insects, such as *D. melanogaster* and *Callinectes sapidus*, MTs are expressed principally in the larval midgut [14], [36]. Durliat *et al* (1995) and Hensbergen *et al* (2000) have reported that the insect gut is the main organ for MT expression in both *D. melanogaster* and *Orchesella cincta* [37], [38] because the gut plays key roles in food absorption, water uptake and waste expulsion. Similarly, metals and other exogenous chemicals can enter the body through the digestive tract during the ingestion of food and water [39]. Surprisingly, MT expression levels were relatively lower in the gut than in the nervous system (brain and optic lobe) in *O. chinensis*. Differing patterns of MT expression may occur according to particular insect species, stages, habitat conditions and dietary habits. Although no studies have focused on MT expression in the nervous system, it is possible that these proteins are highly expressed in the nervous systems of other insects. In this study, the MTs were widely expressed throughout the digestive system and highly expressed in the nervous system. Thus, the digestive system was an important region involved in heavy metal detoxification.

The expressions levels of OcMT1 and OcMT2 in the fat bodies were higher compared with those in the other tissues with the exception of the brain and optic lobe. These findings suggest that OcMT1 and OcMT2 can detoxify exogenous chemicals. The higher expression levels of OcMT2 in the ovaries suggest that this MT may be related to the protection of *O. chinensis* reproduction from metal toxicity or from oxidative stress [13]. Similar results have been reported in several studies of crabs and rats [40], [41]. Therefore, we propose that these two MTs may play important roles in the detoxification of exogenous chemicals. Generally, MTs appear to act as multifunctional stress proteins in higher eukaryotes [42].

MTs are stress proteins that bind with metals and regulate the homeostasis of essential trace metals, counteracting the toxic effects of heavy metals such as Cd, Hg and Ag in insects [43]. The expression patterns of the two OcMT genes were evaluated at all developmental stages. The highest expression levels were found in the eggs, which cannot be considered the first target of heavy metals. These results suggest that high MT mRNA expression may be associated with the oxidative stress response. MTs may also be sensitive to the perturbations of the homeostasis of essential metals, such as Cu and Zn, during embryonic development [44]. MTs may also act in the regulation of redox buffering because redox gradients are important during embryonic development [45]. In aquatic organisms, the early embryo–larval stages appear to be highly sensitive to micropollutants, particularly metals [46]. MTs likely remove O^2−^• and •OH simultaneously, especially considering that MTs react with hydroxyl radicals (OH) at approximately 10,000 times faster rates than superoxide dismutase (SOD) in aquatic invertebrates [47], [48]. In mammals, cells containing increased MT levels are protected against heavy metal toxicity and oxidant stress, whereas the decreased expressions of MTs in cell lines or in MT-null mice has been shown to lead to heightened sensitivities to metal balance disorders and oxidative stress [47], [49]. MTs have high affinities for metals and may play special roles in the regulation of cellular metal distribution [50]; thus, they play key roles in the oxidative stress response and metal homeostasis. Further research is needed to fully elucidate the associated underlying mechanisms.

MTs are known to play physiological roles in essential metal chelation, metal homeostasis, heavy metal detoxification [7], [51], the alleviation of several types of abiotic stresses [52], [53], developmental regulation [54], and the scavenging of reactive oxygen species (ROSs) [55]. Studies have demonstrated that MT concentrations increase in *O. chinensis* when the grasshoppers ingest wheat leaves containing heavy metals. However, the crucial roles of MTs in insects have not been properly elucidated due of the difficulties of purifying the native proteins and cloning the MT sequences [56]. In this study, we evaluated the roles of OcMTs using RNAi and recombinant proteins in an *E. coli* expression system.

RNAi is a meaningful tool in functional genetic analyses. We used this technique to achieve a high degree of silencing of the *OcMT* genes by injecting the dsRNAs into the adult hemocoels. The grasshopper mortality increased after the silencing of *OcMT1* and *OcMT2*. These findings suggest that both genes play important roles in the detoxification of the three metals by chelation, which occurs through their Cys-X-Cys and Cys-X-X-Cys motifs. MTs have been considered to be involved primarily in the detoxification of non-essential and excess essential metals by most authors working in the MT field, and these functions have been observed in species ranging from fungi to mammals [57]. For example, *Lumbricus rubellus*, *Jatropha curcas*, and *Perinereis nuntia* all express distinct MT isoforms that have analogous structure-function relationships for metal binding [58]–[60]. Furthermore, MTs can act as scavengers of the free radicals produced by heavy metal stresses [61], [62] and have been reported to be capable of scavenging free oxygen radicals in transgenic mice and in plants [63], [64].

The expression patterns of the recombinant OcMTs in this study suggested that the cells transformed with the recombinant plasmids had higher Cd tolerances, which may have been due to the chelation of Cd and/or the scavenging of the free radicals produced by Cd by the OcMTs. An increased tolerance to Cd, Zn and Cu has been confirmed in transgenic yeast expressing ThMT3 from *Tamarix hispida* [65]. Enhanced tolerance to the heavy metal cadmium in a recombinant strain expressing an MT has also been demonstrated in *Musca domestica* [66]. A similar study has been performed with the biofuel plant *Jatropha curcas* [59].

In summary, we have described two *MT* genes of *O. chinensis* and analyzed their molecular characteristics and expression patterns. The functions of these two MT genes were investigated using RNAi, and changes in the Cd tolerances of the grasshoppers overexpressing these two MT proteins were analyzed using a recombinant MT expression system. Our results provide novel insights into the tissue localizations of MTs in grasshoppers, which were predominantly expressed in the brain and optic lobe and at the egg stage. Further studies of the regulatory roles of OcMTs in the nervous system (brain and optic lobes) are currently underway. However, additional studies are needed to better understand these results. The roles of OcMTs in cellular metal detoxification will be investigated in our next study.

## References

[1] Margoshes M, Vallee BL (1957) A cadmium protein from equine kidney cortex. J Am Chem Soc 79: 4813–4814.

[2] Liang SH, Jeng YP, Chiu YW, Chen JH, Shieh BS, et al (2009) Cloning, expression, and characterization of cadmium-induced metallothionein-2 from the earthworms *Metaphire posthuma* and *Polypheretima elongata* . Comp Biochem Physiol C: Toxicol Pharmacol 149: 349–357. 18834958 10.1016/j.cbpc.2008.09.004

[3] Vasak M (2005) Advances in metallothionein structure and functions. J Trace Elements Med Biol 19: 13–17. 10.1016/j.jtemb.2005.03.003 16240666

[4] Henkel G, Krebs B (2004) Metallothioneins: Zinc, Cadmium, Mercury, and Copper thiolates and selenolates mimicking protein active site features—Structural aspects and biological implications. Chem Rev 104: 801–824. 14871142 10.1021/cr020620d

[5] Coyle P, Philcox JC, Carey LC, Rofe AM (2002) Metallothionein: The multipurpose protein. Cell Molecular Life Sci ence 59: 627–647. 10.1007/s00018-002-8454-2 PMC11337511 12022471

[6] Chaturvedi AK, Mishra A, Tiwari V, Jha B (2012) Cloning and transcript analysis of type 2 metallothionein gene (SbMT-2) from extreme halophyte *Salicornia brachiata* and its heterologous expression in *E.coli* . Gene 499: 280–287. 22441126 10.1016/j.gene.2012.03.001

[7] Sharma1 S, Rais A, Sandhu R, Nel W, Ebadi M (2013) Clinical signifcance of metallothioneins in cell therapy and nanomedicine. International Journal of Nanomedicine 8: 1477–1488. 23620664 10.2147/IJN.S42019 PMC3633583

[8] Capdevila M, Atrian S (2011) Metallothionein protein evolution: a miniassay. J Biol Inorg Chem 16: 977–989. 21633816 10.1007/s00775-011-0798-3

[9] Ruttkay-Nedecky B, Nejdl N, Gumulec J, Zitka O (2013) The role of metallothionein in oxidative stress. International Journal of Molecular Sciences 14: 6044–6066. 23502468 10.3390/ijms14036044 PMC3634463

[10] Trinchella F, Riggio M, Filosa S, Parisi E, Scudiero R (2008) Molecular cloning and sequencing of metallothionein in squamates: New insights into the evolution of the metallothionein genes in vertebrates. Gene 423: 48–56. 18675328 10.1016/j.gene.2008.06.027

[11] Langston WJ, Bebianno MJ, Burt GR (1998) Metal handling strategies in molluscs: Metal metabolism in aquatic environments, chap. 8. Chapman Hall, London, pp. 219–284.

[12] Andrews GK (2000) Regulation of metallothionein gene expression by oxidative stress and metal ions. Biochemical Pharmacology 59: 95–104. 10605938 10.1016/s0006-2952(99)00301-9

[13] Klaassen CD, Liu J, Diwan BA (2009) Metallothionein protection of cadmium toxicity. Toxicology and Applied Pharmacology 238: 215–220. 19362100 10.1016/j.taap.2009.03.026 PMC2740813

[14] Egli D, Yepiskoposyan H, Selvaraj A, Balamurugan K, Rajaram R, et al (2006) A family knockout of all four Drosophila metallothioneins reveals a central role in copper homeostasis and detoxification. Molecular and cellular biology 26 6: 2286–2296. 10.1128/MCB.26.6.2286-2296.2006 PMC1430275 16508004

[15] Hensbergen PJ, Velzen MJM van, Nugroho RA, Donker MH, Nico M, et al (2006) Metallothionein-bound cadmium in the gut of the insect *Orchesella cincta* (Collembola) in relation to dietary cadmium exposure. Comparative Biochemistry and Physiology Part C 125: 17–24. 10.1016/s0742-8413(99)00087-0 11790326

[16] Yoshida M, Saegusa Y, Fukuda A, Akama Y, Owada S (2005) Measurement of radical scavenging ability in hepatic metallothionein of rat using in vivo electron spin resonance spectroscopy. Toxicology 213: 74–80. 15993999 10.1016/j.tox.2005.05.008

[17] Nakano H, Ikenaga S, Aizu T, Kaneko T, Matsuzaki Y, et al (2006) Human metallothionein gene expression is upregulated by beta-thujaplicin: possible involvement of protein kinase C and reactive oxygen species. Biol Pharm Bull 29: 55–59. 16394509 10.1248/bpb.29.55

[18] Yang LF, Hu N, Jiang SS, Zou YZ, Yang J, et al (2014) Heavy metal scavenger metallothionein attenuates ER stress-induced myocardial contractile anomalies: Role of autophagy. Toxicology Letters 225: 333–341. 24440343 10.1016/j.toxlet.2013.12.024 PMC4041391

[19] Mao H, Wang DH, Yang WX (2012) The involvement of metallothionein in the development of aquatic invertebrate. Aquatic Toxicology 110–111: 208–213. 10.1016/j.aquatox.2012.01.018 22343466

[20] Nakamori T, Fujimori A, Kinoshita K, Ban-nai T, Kubota Y, et al (2010) mRNA expression of a cadmium-responsive gene is a sensitive biomarker of cadmium exposure in the soil collembolan *Folsomia candida* . Environmental Pollution 158: 1689–1695. 20022415 10.1016/j.envpol.2009.11.022

[21] Atanesyan L, Gunther V, Celniker SE, Georgiev O, Schaffner W (2011) Characterization of MtnE, the fifth metallothionein member in *Drosophila* . J Biol Inorg Chem 16: 1047–1056. 21870250 10.1007/s00775-011-0825-4

[22] Zhang YP, Sun Ga, Yang ML, Wu HH, Zhang JZ, et al (2011) Chronic accumulation of cadmium and its effects on antioxidant enzymes and malondialdehydein *Oxya chinensis* (Orthoptera:Acridoidea). Ecotoxicology and Environmental Safety 74: 1355–1362. 21435721 10.1016/j.ecoenv.2011.03.002

[23] Zhang YP, Song DN, Wu HH, Yang HM, Zhang JZ, et al (2014) Effect of Dietary Cadmium on the Activity of Glutathione S-Transferase and Carboxylesterase in Different Developmental Stages of the *Oxya chinensis* (Orthoptera: Acridoidea). Environmental Entomology 43 (1): 171–177. 24342000 10.1603/EN13025

[24] Livak KJ, Schmittgen TD (2001) Analysis of relative gene expression data using real time quantitative PCR and the 2-ΔΔCt Ctmethod. Methods 25: 402–408. 11846609 10.1006/meth.2001.1262

[25] Egli D, Selvaraj A, Yepiskoposyan H, Zhang B, Hafen E, et al (2003) Knockout of ‘metal-responsive transcription factor’ MTF-1 in Drosophila by homologous recombination reveals its central role in heavy metal homeostasis. EMBO J 22: 100–108. 12505988 10.1093/emboj/cdg012 PMC140060

[26] Domenech J, Tinti A, Torreggiani A (2007) Biopolymer research trends. In: Nemeth TS (ed) Research progress on metallothioneins: insights into structure, metal binding properties and molecular function by spectroscopic investigations. Nova Science Publishers, Inc., New York, 11–48.

[27] Chung WP, Dewan JC, Walters MA (1991) Models of lysine–cysteine hydrogen bonding in metallothionein: hydrogen bonding between ammonium and benzenethiolate in [(C6H11)_2_NH_2_]^2^[Co(SC_6_H_5_)^4^]. J Am Chem Soc 113: 525–530.

[28] Ren F, Jiang H, Sun J, He L, Li W, et al (2010) Cloning, characterization, expression, and copper sensitivity of the metallothionein-1 gene in the Chinese mitten crab, *Eriocheir sinensis* . Mol Biol Rep 38: 2383–2393. 21082264 10.1007/s11033-010-0372-z

[29] Mayo KE, Warren R, Palmiter RD (1982) The mouse metallothionein-I gene is transcriptionally regulated by cadmium following transfection into human or mouse cells. Cell 29: 99–108. 6955027 10.1016/0092-8674(82)90094-0

[30] Aras MA, Aizenman E (2011) Redox regulation of intracellular Zinc: molecular signaling in the life and death of neurons. Antioxidants and redox signaling 15 (8): 2249–2263. 20849376 10.1089/ars.2010.3607 PMC3166180

[31] Thirumoorthy N, Shyam Sunder A, Manisenthil Kumar K, Senthil Kumar M, Ganesh G, et al (2011) A Review of Metallothionein isoforms and their role in pathophysiology. World Journal of Surgical Oncology 9: 54. 21599891 10.1186/1477-7819-9-54 PMC3114003

[32] Manso Y, Adlard PA, Carrasco J, Vasak M, Hidalgo J (2011) Metallothionein and brain inflammation. J Biol Inorg Chem 16(7): 1103–1113. 21678079 10.1007/s00775-011-0802-y

[33] Hidalgo J, Aschner M, Zatta P, Vasak M (2001) Roles of the metallothionein family of proteins in the central nervous system. Brain Research Bulletin 55 (2): 133–145. 11470309 10.1016/s0361-9230(01)00452-x

[34] West AK, Hidalgo J, Eddins D, Levin DE, Aschner M (2008) Metallothionein in the central nervous system: Roles in protection, regeneration and cognition. Neuro Toxicology 29: 489–503. 10.1016/j.neuro.2007.12.006 PMC2486367 18313142

[35] Levin ED, Perraut C, Pollard N, Freedman JH (2006) Metallothionein expression and neurocognitive function in mice. Physiology & Behavior 87: 513–518. 16430929 10.1016/j.physbeh.2005.11.014

[36] Brouwer M, Syring R, Hoexum-Brouwer T (2002) Role of a copper-specific metallothionein of the blue crab, *Callinectes sapidus*, in copper metabolism associated with degradation and synthesis of hemocyanin. J Inorg Biochem 88: 228–239. 11803044 10.1016/s0162-0134(01)00381-6

[37] Durliat M, Bonneton F, Boissonneau E, Andre M, Wegnez M (1995) Expression of metallothionein genes during the postembryonic development of *Drosophila melanogaster* . Biometals 8: 339–351. 7580054 10.1007/BF00141608

[38] Hensbergen PJ, van Velzen MJ, Nugroho RA, Donker MH, van Straalen NM (2000) Metallothionein-bound cadmium in the gut of the insect *Orchesella cincta* in relation to dietary cadmiumexposure. Comp BiochemPhysiol C Toxicol Pharmacol 125: 17–24. 10.1016/s0742-8413(99)00087-0 11790326

[39] Molto E, Bonzon-Kulichenko E, del Arco A, Lopez-Alanon DM, Carrillo O, et al (2005) Cloning, tissue expression and metal inducibility of an ubiquitous metallothionein from *Panulirus argus* . Gene 361: 140–148. 16185828 10.1016/j.gene.2005.07.026

[40] Xiang DF, Zhua JQ, Jin S, Hu YJ, Tan FQ, et al (2013) Expression and function analysis of metallothionein in the testis of *Portunus trituberculatus* exposed to cadmium. Aquatic Toxicology 140–141: 1–10. 10.1016/j.aquatox.2013.05.004 23747547

[41] Maremanda KP, Khan S, Jena G (2014) Zinc protects cyclophosphamide-induced testicular damage in rat: involvement of metallothionein, tesmin and Nrf2. Biochemical and Biophysical Research Communications 41 (3): 591–596. 10.1016/j.bbrc.2014.02.055 24565835

[42] Andrews GK (2000) Regulation of metallothionein gene expression by oxidative stress and metal ions. Biochemical Pharmacology 59: 95–104. 10605938 10.1016/s0006-2952(99)00301-9

[43] Viarengo A, Burlando B, Cavaletto M, Marchi B, Ponzano E, et al (1999) Role of metallothionein against oxidative stress in the mussel *Mytilus galloprovincialis* . Am J Physiol 277: 1612–1619. 10.1152/ajpregu.1999.277.6.R1612 10600906

[44] Roesijadi G, Hansen KM, Unger M (1996) Cadmium-induced metallothionein expression during embryonic and early larval development of the mollusc *Crassostrea virginica* . Toxicol Appl Pharmacol 140: 356–363. 8887452 10.1006/taap.1996.0231

[45] Amiard JC, Amiard-Triquet C, Barka S, Pellerin J, Rainbow PS (2006) Metallothioneins in aquatic invertebrates: their role in metal detoxication and their use as biomarkers. Aquat Toxicol 76: 160–202. 16289342 10.1016/j.aquatox.2005.08.015

[46] Martin M, Osborn KE, Billig P, Glickstein N (1981) Toxicities of ten metals to *Crassostrea gigas* and *Mytilus edulis* embryos and Cancer magister larvae. Mar Pollut Bull 12: 305–308.

[47] Ruttkay-Nedecky B, Nejdl L, Gumulec J, Zitka O, Masarik M, et al (2013) The role of metallothionein in oxidative stress. International Journal Molecular Sciences 14: 6044–6066. 10.3390/ijms14036044 PMC3634463 23502468

[48] Pan L, Zhang H (2006) Metallothionein, antioxidant enzymes and DNA strandbreaks as biomarkers of Cd exposure in a marine crab, *Charybdis japonica* . Comp Biochem Physiol C: Toxicol Pharmacol 144: 67–75. 16908220 10.1016/j.cbpc.2006.06.001

[49] Moulis Jean-Marc (2010) Cellular mechanisms of cadmium toxicity related to the homeostasis of essential metals. Biometals 23: 877–896. 20524046 10.1007/s10534-010-9336-y

[50] Banci L, Bertini I, Ciofi-Baffoni S, Kozyreva1 T, Zovo K, et al (2010) Affinity gradients drive copper to cellular destinations. Nature 465: 645–648. 20463663 10.1038/nature09018

[51] Usha B, Venkataraman G, Parida A (2009) Heavy metal and abiotic stress inducible metallothionein isoforms from *Prosopis juliflora* (SW) D.C. show differences in binding to heavy metals in vitro. Mol Genet Genomics 281: 99–108. 19015881 10.1007/s00438-008-0398-2

[52] Yang Z, Wu YR, Li Y, Ling HQ, Chu C (2009) OsMT1a, a type 1 metallothionein, plays the pivotal role in zinc homeostasis and drought tolerance in rice. Plant Mol Biol 70: 219–229. 19229638 10.1007/s11103-009-9466-1

[53] Gonzalez-Mendoza D, Moreno AQ, Zapata-Perez O (2007) Coordinated responses of phytochelatin synthase and metallothionein genes in black mangrove, *Avicennia germinans*, exposed to cadmium and copper. Aquat Toxicol 83: 306–314. 17582515 10.1016/j.aquatox.2007.05.005

[54] Guo WJ, Bundithya W, Goldsbrough PB (2003) Characterization of the Arabidopsis metallothionein gene family: tissue-specific expression and induction during senescence and in response to copper. New Phytol 159: 369–381. 10.1046/j.1469-8137.2003.00813.x 33873353

[55] Akashi K, Nishimura N, Ishida Y, Yokota A (2004) Potent hydroxyl radical scavenging activity of drought-induced type-2 metallothionein in wild watermelon. Biochem Biophys Res Commun 323: 72–78. 15351703 10.1016/j.bbrc.2004.08.056

[56] Freisinger E (2011) Structural features specific to plant metallothioneins. J Biol Inorg Chem 16: 1035–1045. 21688177 10.1007/s00775-011-0801-z

[57] Carpene E, Andreani G, Isani G (2007) Metallothionein functions and structural characteristics. Journal of Trace Elements in Medicine and Biology 21: 35–39. 18039494 10.1016/j.jtemb.2007.09.011

[58] Sturzenbaum SR, Winters C, Galay M, Morgan AJ, Kille P (2001) Metal ion trafficking in earthworms identification of a cadmium-specfic metallothionein. The Journal of Biological Chemistry 276: 34013–34018. 11418603 10.1074/jbc.M103605200

[59] Mudalkar S, Golla R, Sengupta D, Ghatty S, Reddy AR (2014) Molecular cloning and characterisation of metallothionein type 2a gene from *Jatropha curcas* L., a promising biofuel plant. Mol Biol Rep 41: 113–124. 24190491 10.1007/s11033-013-2843-5

[60] Won EJ, Rhee JS, Ra K, Kim KT, Au DWT, et al (2012) Molecular cloning and expression of novel metallothionein(MT) gene in the polychaete *Perinereis nuntia* exposed to metals. Environ Sci Pollut Res 19: 2606–2618. 10.1007/s11356-012-0905-1 22828888

[61] Sato M, Bermner I (1993) Oxygen free radicals and metallothionein. Free Rad Bio 14: 325–337. 10.1016/0891-5849(93)90029-t 8458590

[62] Sharma S, Ebadi M (2014) Significance of metallothioneins in aging brain. Neurochemistry International 65: 40–48. 24389356 10.1016/j.neuint.2013.12.009

[63] Sato M, Kondoh M (2002) Recent studies on metallothionein: protection against toxicity of heavy metals and oxygen free radicals. The Tohoku Journal of Experimental Medicine 196 (1): 9–22. 12498322 10.1620/tjem.196.9

[64] Dietz KJ, Baier M, Kramer U (1999) Free radicals and reactive oxygen species as mediators of heavy metal toxicity in plants. Heavy Metal Stress in Plants 73–97.

[65] Yang J, Wang Y, Liu G, Yang C, Li C (2011) *Tamarix hispida* metallothionein-like ThMT3, a reactive oxygen species scavenger, increases tolerance against Cd^2+^, Zn^2+^, Cu^2+^ and NaCl in transgenic yeast. Mol Biol Rep 38 (3): 1567–1574. 20835888 10.1007/s11033-010-0265-1

[66] Tang T, Huang DW, Zhang D, Wu YJ, Murphy RW, et al (2011) Identification of two metallothionein genes and their roles in stress responses of *Musca domestica* toward hyperthermy and cadmium tolerance. Comparative Biochemistry and Physiology Part B 160: 81–88. 10.1016/j.cbpb.2011.06.008 21762786

